# Analysis of Starch Structure and Pasting Characteristics of Millet Thick Wine during Fermentation

**DOI:** 10.3390/foods12091840

**Published:** 2023-04-28

**Authors:** Lixia Wang, Huanyu Ming, Qi Chen, Huayin Pu, Xiaoping Li, Peng Wang, Lihui Zhu, Jing Yan, Haoran Liu

**Affiliations:** 1 College of Life Sciences and Food Engineering, Shaanxi Xueqian Normal University, Xi’an 710100, China; 2 College of Food Engineering and Nutritional Science, Shaanxi Normal University, Xi’an 710119, China; 3 School of Food Science and Engineering, Shaanxi University of Science and Technology, Xi’an 710021, China

**Keywords:** MTW, starch, structure, features

## Abstract

Starch is the main substrate in millet thick wine (MTW). In order to control the fermentation process of MTW, it is critical to monitor changes in the starch structure and physicochemical characteristics during the fermentation of MTW. In the present study, the structural characteristics of MTW starch were analyzed by scanning electron microscopy (SEM), confocal laser scanning microscopy (CLSM), X-ray diffraction (XRD), Fourier transform infrared spectroscopy (FTIR) and rapid viscosity analysis (RVA). The results of SEM and CLSM showed that large starch granules in MTW swelled, developed cavities, and ruptured or even vanished with the prolongation of the fermentation time, whereas the size and shape of small starch granules barely changed, only falling off the pomegranate-seed-like aggregates. With the increase in fermentation time, the relative crystallinity of starch in MTW gradually increased. In addition, the short-range ordered structures underwent complex changes. Changes in the starch morphology and ordered structure led to an increase in the peak viscosity time and the initial gelatinization temperature. The present results reveal the beneficial effect of fermentation on MTW processing and suggest its potential applications in other millet-based fermented products.

## 1. Introduction

Proso millet (*Panicum miliaceum* L.), an important member of the millet family, is the seed of the annual herbaceous crop *Panicum* and *Sect. Panicum* [1]. Millet is one of the oldest and most important domesticated crops in China and other developing countries [2,3,4]. Archaeological studies have proved that China began to cultivate and domesticate wild millet at least 10,000 years ago [2,3,4,5,6]. Although its routes of dispersal are unclear, millet became a staple food in semi-arid areas of East Asia and the entirety of Eurasia [2]. It is still an important food in these areas today [5,6,7,8,9]. Millet has a high nutritional value, with starch content of about 60–70%, up to 11% protein and dietary fiber ranging from 2 to 7% [10,11]. It is also a good source of minerals, such as calcium, iron and magnesium, and essential amino acids, such as methionine and cysteine. In addition, millet is rich in phenolic acids, melanoids, flavonoids and tannins, the contents of which are several times higher than those of other staple grains [12]. Studies have shown that these phytochemicals have antioxidant, anti-cancer, anti-diabetic, anti-ulcer, anti-bacterial, anti-cardiovascular, anti-inflammatory and wound-healing properties [13,14,15]. Therefore, millet is processed into many kinds of food. In particular, fermented foods such as millet wine are very popular in China.

**Figure 1:**
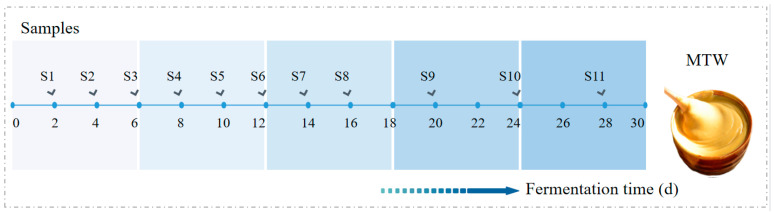
Information diagram of MTW samples.

Fermentation is a preparation technology that uses beneficial microorganisms to give foods new flavors, aromas and textures. Traditional fermented food has a long history in China, dating back to 8000 BC [16]. As the earliest fermented food in China, rice wine is a treasure of Chinese culture and a precious scientific heritage. Meanwhile, as one of the three ancient wines in the world, along with wine and beer, rice wine is popular among consumers because of its unique flavor, delicate taste and appropriate alcohol content [17]. Nowadays, rice wine, known as the state banquet wine, has become an important part of Chinese liquor consumption and plays an important role in the daily lives of Chinese people [18]. There are multitudinous types of Chinese rice wine, with differences in the raw material, starter, ingredients and method of production [19]. On the basis of its appearance, Chinese rice wine can be divided into two types. One is clear and transparent because the solid substances (mash) in the fermentation liquor are filtered by pressing after fermentation; this type is called Chinese Huangjiu or Chinese yellow wine [19]. The other is opaque, in the form of rice paste (as shown in Figure 1), and traditionally produced from millet; this type is called Choujiu or millet thick wine (MTW). Huangjiu is the most typical Chinese rice wine and has been widely studied [19]. Traditional Huangjiu can be produced with glutinous rice (northern Huangjiu) or coarse cereals, such as buckwheat, sorghum, oats and millet [17,19,20,21]. Due to its unique flavor and high nutritional value, millet Huangjiu has gradually attracted the attention of researchers in recent years [17,19,20,21]. Huang et al. [16] identified the key odorants of broomcorn millet (*Panicum miliaceum* L.) and the succession patterns of aroma components during the brewing process of broomcorn millet Huangjiu [17]. Yan et al. [21] studied the changes in microbial diversity and flavor compounds during the fermentation of millet Huangjiu. In addition, the physicochemical and structural properties of red millet during pretreatment (soaking and steaming) were also reported [21]. However, few studies have reported on the processing of millet thick wine and its changes during fermentation.

In northern Shaanxi, millet is traditionally processed into millet thick wine (MTW), which is a very popular food and is also a very important form of processed and consumed local millet. For processing millet thick wine, whole millet grains are crushed, steamed, added to the starter (wheat koji) and fermented. The fermentation liquid and solid substances are retained in the product because no filtering is required after fermentation, which is named the solid-state fermentation of whole grains. During the fermentation process of MTW, its protein and starch can be decomposed into various amino acids, polypeptides, low-molecular-weight sugars, etc., that are easily absorbed and utilized by the human body [20,21]. Starch, as the main substrate in millet, undergoes complicated physical and chemical changes during the fermentation process of MTW, and some unfermented starch may remain in MTW, which might have a great impact on the quality of millet thick wine. However, changes in the structural and physicochemical characteristics of starch in MTW during fermentation have rarely been investigated. Therefore, several methods, including scanning electron microscopy (SEM), confocal laser scanning microscopy (CLSM), rapid viscosity analysis (RVA), X-ray diffraction (XRD) and Fourier transform infrared spectroscopy (FTIR), were used to explore the effects of different fermentation times on the structural and physicochemical properties of MTW starch. This study is expected to provide a theoretical basis for better revealing the beneficial effects of fermentation on MTW and lay a foundation for further exploring the health effects of MTW in the future.

## 2. Materials and Methods

### 2.1. Materials and Chemical Reagents

Millet and millet thick wine were provided by Xing’hua Wine Industry (Yi’chuan, Shaanxi, China).

Fluorescein isothiocyanate (FITC) was purchased from Fuzhou Feijing Biotechnology Co. LTD (Fuzhou, Fujian, China). Rhodamine B (Rhodamn) was purchased from Tianjin Zhiyuan Chemical Reagent Co. LTD (Tianjin, Dongli, China). Acetone, glycerin and other solvents, chemicals and reagents were purchased from Xi’an Jingbo Chemical Reagent Company (Xi’an, Shaanxi, China). All chemicals were analytically pure unless otherwise stated.

### 2.2. MTW and Sample Preparation

MTW: After grinding and sieving the samples through a 100-mesh screen, millet (100 Kg) was added to a mixing tank (500 L, Zigong Tianxiang Ceramics Co., Ltd., Tongsi Town, China), and then water (at a ratio of 2:1, *w*/*w*) was added to the millet and stirred to mix it evenly. The mixture was put into a wooden steamer and steamed at atmospheric pressure for 2 h and then cooled to about 20 °C. Wheat koji (4.5%, based on millet weight) was added, put into the fermentation tank (ZC300, Chaoxia Technology Co., Ltd., Zhejiang, China), stirred evenly, fermented at about 18 °C, stirred and vented every day during fermentation. Samples of about 200 g were taken every two days from fermentation day 0 to day 32 of fermentation. The samples were packed into boxes, sealed and stored in a low-temperature refrigerator at −40 °C for later analysis. The sample information in this study is shown in Figure 1.

### 2.3. Starch Extraction from MTW and Millet

Starch was extracted from MTW and millet based on the method described by Li et al. [22]. First, 150 g of MTW was taken, and a 0.3 g/100 mL NaOH solution was added at a ratio of solid to liquid of 1:3. The mixture was placed in a shaking bed, shaken for 3 h and centrifuged for 10 min at 4000 r/min. The supernatant was discarded, and the surface impurities were scraped off. Then, distilled water was added to the precipitate and washed repeatedly until no impurities were observed. The starch from raw millet can be observed to separate into two layers during the extraction process. The upper starch layer is white, called white starch, and the lower starch layer is yellow, called yellow starch. The starch was collected, and freeze-dried starch was passed through a 200-mesh screen, sealed and stored for later use.

### 2.4. Scanning Electron Microscopy (SEM)

SEM was used to observe the surface morphology of MTW starch with reference to the method reported by Wei et al. [23], with some modifications. The MTW starch samples were sputtered with gold powder by a Cressington 108 auto sputter coater (Cressington Scientific Instruments, Watford, UK) at a current of 40 mA for 120 s. The surface morphology of starch granules was observed by using an environmental scanning electron microscope (Quanta 200, FEI Company, Hillsboro, OR, USA) at an accelerating voltage of 30 kV and magnification of 200× (white scale bar = 400 μm), 2500× (white scale bar = 20 μm), 10,000× (white scale bar = 10 μm) and 12,000× (white scale bar = 10 μm).

### 2.5. Confocal Laser Scanning Microscopy (CLSM)

Fluorescein isothiocyanate isomer (FITC) was dissolved in acetone to produce a 0.5 mg/mL FITC dye solution with reference to the method reported by Chen et al. [24], with some modifications. The appropriate amount of Rhodamine B was dissolved in distilled water and prepared as a 1 mg/mL Rhodamine B dye solution.

Dyeing method: First, 40 mg of millet and MTW starch powder were separately dispersed into 1 mL of distilled water to form an emulsion, and then the emulsion was mixed with 2 mL of mixed dye containing 0.1% FITC dye and 0.1% Rhodamine B dye. The emulsion was dyed overnight at room temperature without light and then rinsed with pH7.4 PBS buffer solution until the lotion was colorless, and the lower part was precipitated. The sample was placed on a concave glass slide coated with glycerin, which was then covered.

Observation Method: The internal structure and integrity of starch were observed by using a confocal laser scanning microscope equipped with an inverted microscope (Olympus FV1200, Tokyo, Japan). The slides were placed on a laser confocal microscope (CLSM), and a 40× objective lens with a resolution of 512 × 512 was used. The laser wavelengths/fluorescence emission wavelengths of FITC and Rhodamine B were 488/518 nm and 543/625 nm, respectively. Each sample was photographed at 40× for 3 parallel images.

### 2.6. X-ray Diffraction (XRD)

According to the method reported by Han et al. [25], an X-ray diffractometer (D8 Advance, Bruker Inc., Falkenried, Karlsruhe, Germany) was used to analyze the semi-crystalline structure of MTW starch. According to the method described by Chen et al. [26], the pre-balanced millet and MTW starch were laid flat in the sample pool and then put into the sample table of the X-ray diffractometer for detection. A monochromatic Cu-Kα ray with a wavelength of 0.1542 nm was selected. The type of optical tube is a Cu target, ceramic X-ray tube, and the optical tube power is 2.2 kW; optical encoder technology and a double-positioning stepper motor were used with a LynxEye array detector. The test conditions were as follows: tube pressure 40 kV, tube flow 40 mA, scanning area 2θ = 4~40°, step angle 0.0001°, angle reproducibility 0.0001° and continuous scanning time 15 s.

### 2.7. Fourier Transform Infrared Spectroscopy (FTIR)

Referring to the method of Bai et al. [27], a Fourier transform infrared spectrometer (Bruker GmBH, Ettlingen, Germany) was used to analyze the short-range ordered structure of MTW starch. The potassium bromide (KBr) tablet method was used, and the sample was fully mixed with starch in a 1% proportion, ground, tableted and tested. The detection conditions were as follows: full wavelength 4000~400 cm^−1^ was selected as the scanning wave number (4000~1300 cm^−1^ was the functional group region, and 1300~800 cm^−1^ was the fingerprint region), and the resolution was 4 cm^−1^.

### 2.8. Pasting Characteristics

According to the method reported by Zhang et al. [28], a rapid viscosity analyzer (TechMaster, Perten Ruihua Scientific Instrument Co., Ltd., Beijing, China) was used to analyze the gelatinization characteristics of the starch in MTW. A starch emulsion of 28 G 5% (m/m) was accurately prepared, and the corn millet starch was placed in the special aluminum RVA box for measurement in the rapid viscosity analyzer. The heating and cooling cycle procedure is as follows: When the temperature rose to 50 °C, the time started and the temperature was maintained for 1 min. The temperature was raised to 95 °C at 12 °C/min, and heat was maintained for 5 min. Then, it was cooled at 12 °C/min to 50 °C and maintained at this temperature for 2 min. The initial rotation rate of stirring was 960 r/min, and after 10 s, the rotation speed increased to 160 r/min.

## 3. Results and Discussion

### 3.1. Morphology by Scanning Electron Microscopy (SEM)

**Figure 2:**
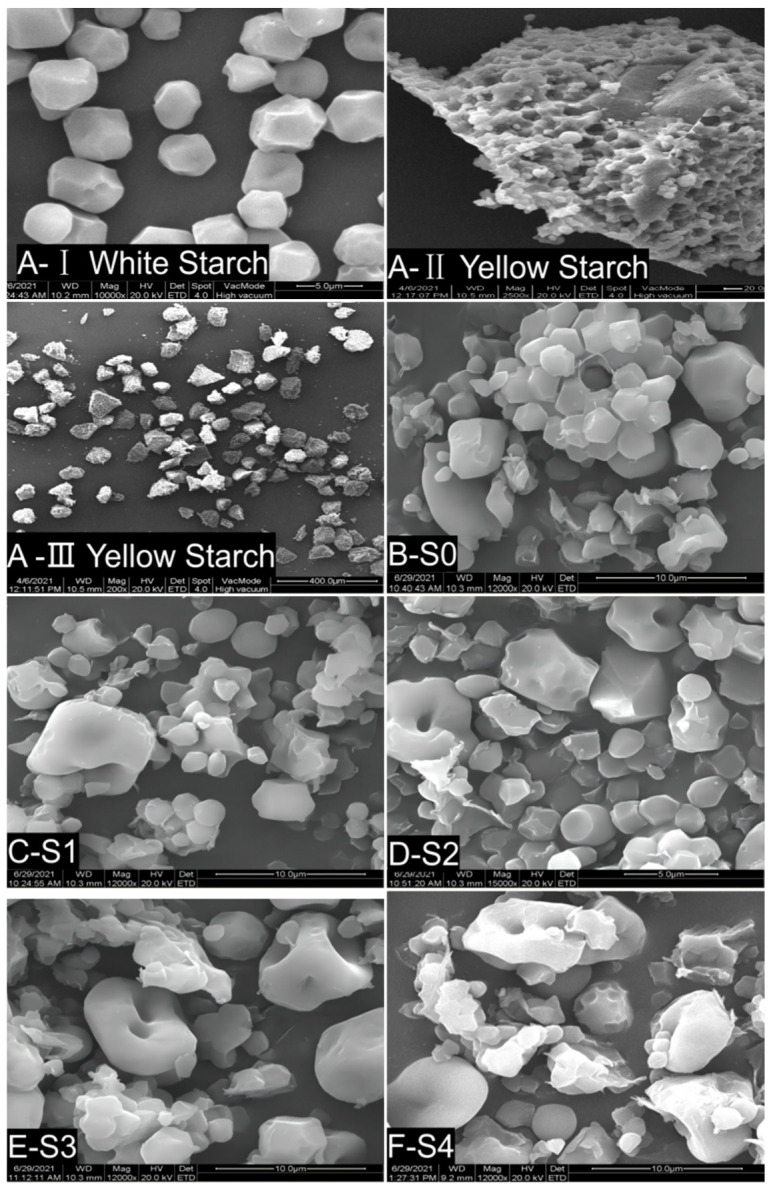

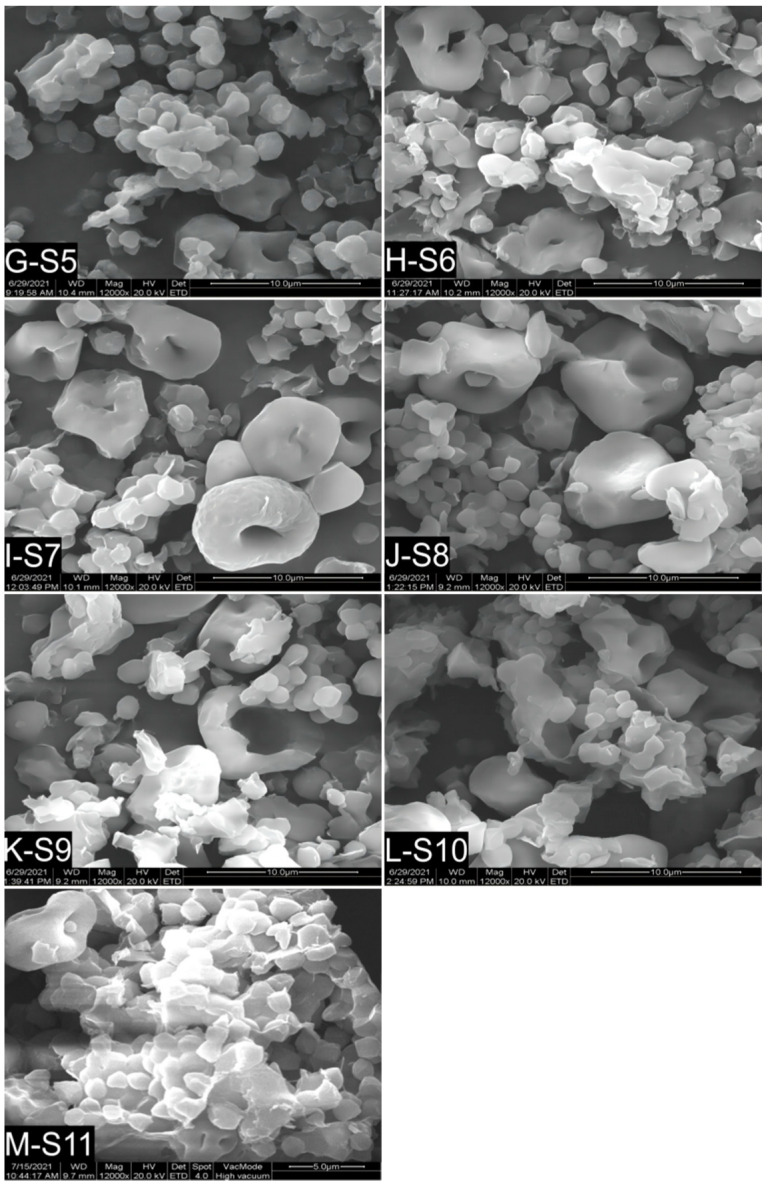
Surface morphology photographs of millet and MTW starch on different fermentation days under scanning electron microscope. Note: (**A**-**I**) White starch from raw millet grains, (**A-I**–**A-III**) yellow starch from millet at 10,000×, 2500× and 200×. (**B-S0**–**M-S11**) MTW starch after fermentation for 0, 2, 4, 6, 8, 10, 12, 14, 16, 20, 24 and 28 days at 12,000×, respectively.

Figure 2 shows the surface morphological structure of starch from millet and MTW in different fermentation stages. There are two kinds of millet starch, which can be extracted from raw millet. According to the color of the extracts, they are respectively called white starch and yellow starch. As can be seen in Figure A-I, white starch consists of spherical or angular spherical granules, about 3~5 μm in diameter, and their surfaces are smooth and have visible edges and corners: this is called large granular starch. Yellow starch (Figure 2A-II) is an aggregation of small starch granules with a spherical or ovoid shape, about 1 to 2 μm in diameter. The surfaces of the small starch granules have no edges or corners, and their shape is nearly spherical and closely packed together like pomegranate seeds. Small starch granules clustered into lumpy irregularities, and their diameters were about 100 to 200 μm.

Figure 2B shows the surface morphology of millet starch after cooking, which clearly reveals the differences between the two kinds of millet starch. Meanwhile, it can be seen that steaming causes some large starch granules to lose their morphological structure and the aggregation of small starch granules to become loose. After fermentation, significant changes in the morphological characteristics of the two kinds of millet starch can be observed (Figure 2C–M). On the second day of fermentation, large starch grains appeared swollen, contained cavities, and had ruptured or even vanished. The size and shape of small starch granules were almost unchanged, but small starch granules gradually fell from the pomegranate-seed-like aggregates. With the prolongation of fermentation, the change becomes more apparent. After fermentation for 20 days (Figure 2K), large integral starch granules can hardly be observed, and small starch granules are dispersed individually. The present results indicate that large starch granules in millet can be degraded, digested and utilized by microorganisms and are attributable to fermentable starch, while small starch granules cannot be degraded and are attributable to non-fermentable starch.

The present observation results of millet starch granules and their surface morphologies are consistent with previous reports [6,10,29,30], which found that polyhedral shapes with smooth edges and a spherical shape dominate the millet starch, and almost 70% of starch granules are unfissured. The sizes of the smallest to largest starch granules varied from 0.3 to 17 μm. The impact of fermentation on the structure and properties of starch has been widely reported [23]. Fermentation significantly altered the structure of millet starch granules, as their surfaces apparently eroded after fermentation by yeast [31]. The surfaces of starch granules from glutinous Proso millet [32], wheat [23] and rice [33] had cracks and holes after spontaneous fermentation and *Lactobacillus plantarum* (*LP*) fermentation. Paixão e Silva Giselle de Lima et al. [31] observed that the surfaces of starch granules were sunken during sweet potato fermentation. They speculated that the cracks and holes in starch granules were corroded by metabolites of fermentation bacteria. In addition, the pits of starch granules during fermentation may also be caused by the degradation of starch granules. In this study, wheat *koji* (a mixture of yeast and filamentous fungus (*Aspergillus oryzae*)) was added to the millet to promote fermentation. Enzymes such as a-amylase, glucoamylase and protease are biosynthesized by microorganisms, which catalyze the decomposition of starch and protein [34]. Yeast facilitates alcohol fermentation, and alcohol can also cause changes in the starch granule structure. Farrag et al. [35] found that aqueous-alcoholic treatment can be applied to prepare donut-shaped starch microparticles. They indicated that when ethanol is added to starch, inclusion complexes are formed, and the granules shrink because of dehydration. Therefore, the morphological changes in millet starch may be due to the dual effects of enzymes secreted by microorganisms, and their metabolites, such as acid and alcohol products, attack millet starch granules and promote starch degradation during fermentation. The small starch granules cannot be fermented by microorganisms, indicating that their structure may be tight and orderly and have good anti-digestion performance. Therefore, these small starch granules may be attributable to slowly digested or resistant starch, which can endow MTW with good functional characteristics.

### 3.2. Morphology by Confocal Scanning Laser Microscopy (CLSM)

**Figure 3:**
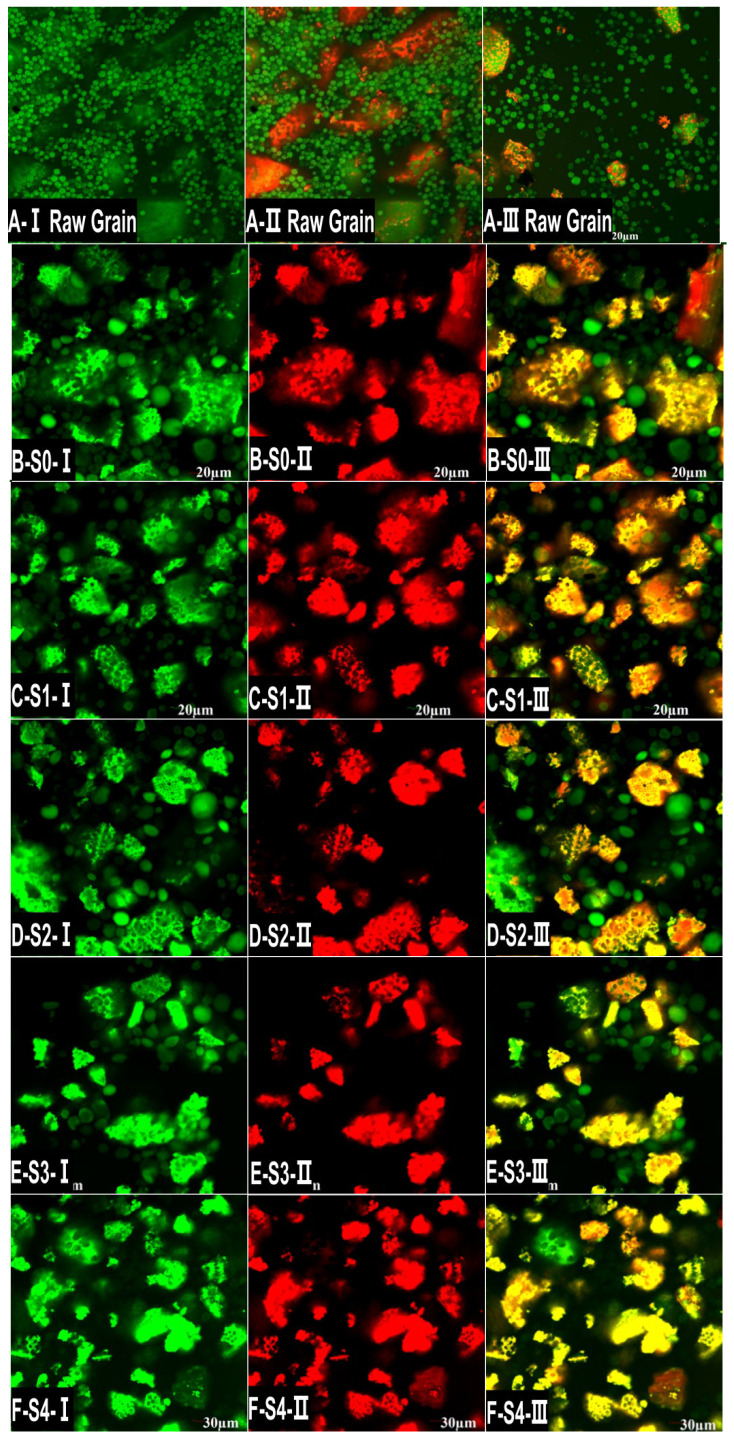

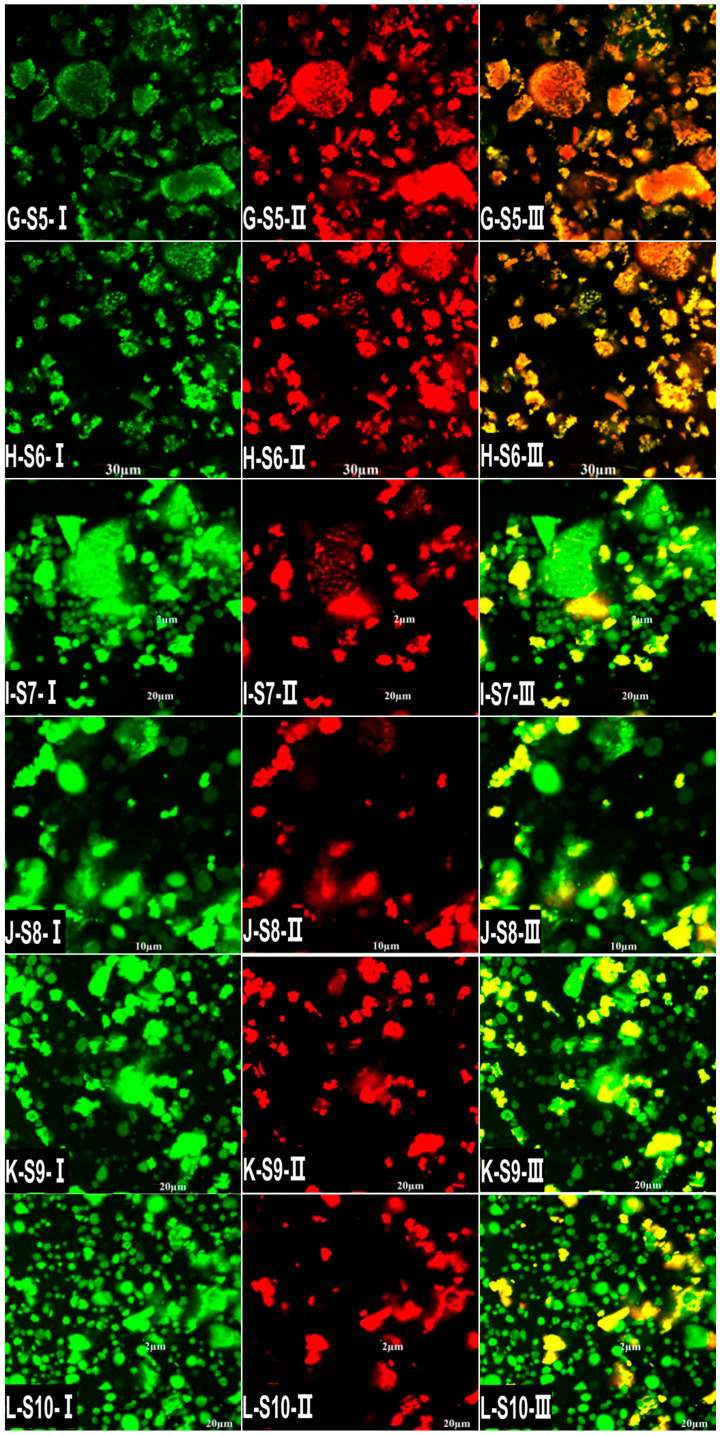

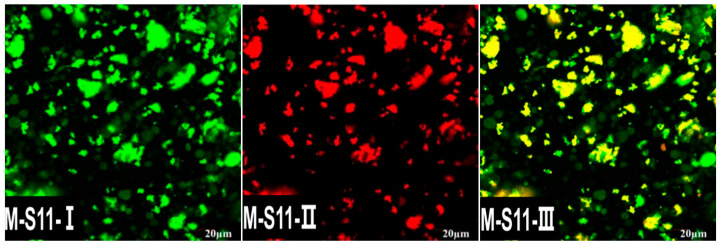
CLSM photographs of MTW starch on different fermentation days. Note: (**A-Ⅰ**–**A-Ⅲ**) refer to raw millet starch granules, and (**B-S0**–**M-S11**) refer to MTW starch after fermentation for 0, 2, 4, 6, 8, 10, 12, 14, 16, 20, 24 and 28 days, respectively. (**Ⅰ**–**Ⅲ)** refers to the staining of MTW by FITC and Rhodamine B, in which the protein is colored, the starch is colored, and the protein and starch are colored together.

Confocal laser scanning microscope photographs of starch from millet and MTW on different fermentation days are shown in Figure 3. According to the color characteristics, the green particles are starch, and the red area is protein. Figure 3A shows that there are two kinds of millet starch. One is large starch granules with a diameter of 3–5 μm. The other is small granules of starch, about 1 to 2 μm in diameter, packed together like pomegranate seeds and intertwined with proteins. The present findings are consistent with the results of SEM and previous studies. It can also be seen that the surface of starch granules, especially aggregates of small starch particles, is coated with proteins. At first, the encapsulation of proteins is dense and integrated in the raw millet (Figure 3A). Naguleswaran et al. [36] observed the morphology of rye and corn starch under a CLSM and found that the surface of starch granules was rich in protein. After steaming, the encapsulation of proteins became loose and porous, which may be caused by water absorption and the expansion of proteins. After fermentation, large protein packages gradually became small pieces, and the small pieces became smaller and smaller as the fermentation time was prolonged, which may be attributed to the degradation of proteins. Numerous studies have pointed out that the proteins in raw materials are broken down and form peptides and amino acids by microorganisms from koji during the production of Chinese rice wine [37,38,39]. Figure 3B–M show that the large starch granules and the encapsulation of proteins from millet first expanded and then burst and disappeared, and the pomegranate-like aggregates gradually decomposed and became smaller after cooking and fermentation. This is consistent with the SEM observations.

### 3.3. Crystalline Structure and Relative Crystallinity

**Figure 4:**
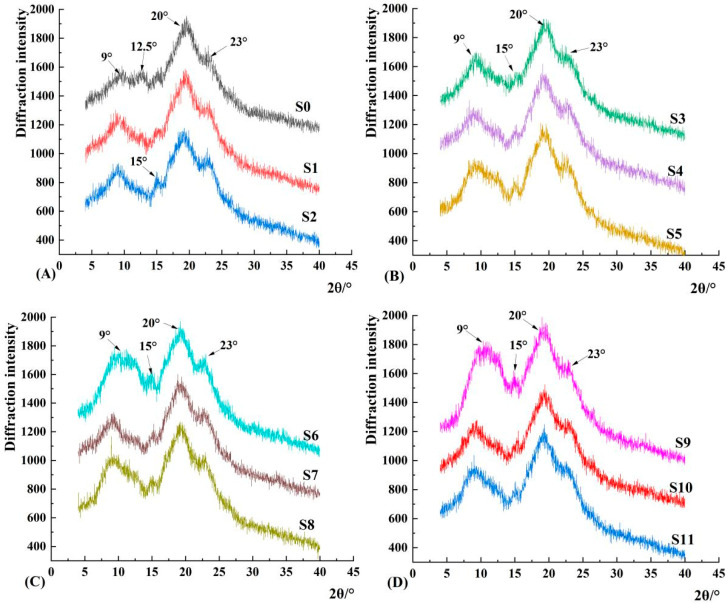
X-ray diffraction patterns of MTW starch at different fermentation days. Note: (**A**) MTW starch after fermentation for 0, 2 and 4 days; (**B**) MTW starch after fermentation for 6, 8 and 10 days; (**C**) MTW starch after fermentation for 12, 14 and 16 days; (**D**) MTW starch after fermentation for 20, 24 and 28 days.(**S0**–**S11**) refer to MTW starch after fermentation for 0, 2, 4, 6, 8, 10, 12, 14, 16, 20, 24 and 28 days, respectively.

XRD was used to analyze the long-range ordered structure of the starch samples, which can reveal the crystallization type and relative crystallinity of starch [40]. Figure 4 shows the X-ray diffraction patterns of starch from millet and MTW in different fermentation stages. Figure 4 shows that millet starch and MTW starch in different fermentation stages have diffraction peaks at 9°, 12.5°, 15°, 20° and 23°, which is a typical A-type crystal structure, which is consistent with the conclusion that cereal starch is mostly A-type crystal [41]. It can be seen in Figure 4 that fermentation did not change the crystal type of millet starch, but the diffraction intensity of 12.5° gradually weakened, while the diffraction intensities at 9° and 20° gradually increased and became increasingly sharper with the increase in fermentation days, indicating that fermentation caused some changes in the crystalline structure of millet starch. The analysis results of the relative crystallinity of starch from millet and MTWs in different fermentation periods are shown in Table 1. Table 1 shows that the relative crystallinity of millet starch first increases, then decreases and increases again with the increase in fermentation time, and the relative crystallinity of all fermented starch is greater than that of raw starch. QiLai et al. [42] studied the fermentation of rice starch and obtained similar results. They speculated that the enzymolysis of starch molecules could enhance crystallinity. In addition, Waleed AL Ansi et al. [43] believed that the increase in the relative crystallinity of highland barley starch was due to the hydrolysis of amorphous regions of starch granules during fermentation. The results of SEM and CLSM showed that the large starch granules in the millet starch gradually disintegrated, and the small starch granules dispersed from the aggregates to become the main body with the prolongation of fermentation time. The present findings indicate that the large starch granules in the millet may have a lower relative crystallinity, while the small starch granules have a more ordered structure. Of course, the change in relative crystallinity may also be due to the decomposition of amorphous areas in large starch grains, resulting in a relative increase in crystallized areas. However, generally speaking, after fermentation, millet starch has a more orderly long-term structure, which is consistent with the gelatinization characteristics of fermented starch.

### 3.4. Short-Range Ordered Structures

**Figure 5:**
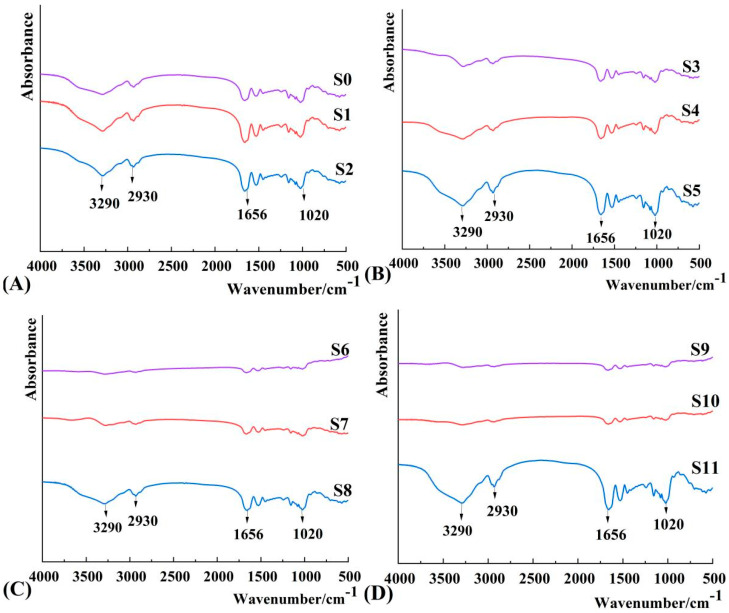
Fourier transform infrared analysis of MTW starch on different fermentation days. Notes: (**A**) MTW starch after fermentation for 0, 2 and 4 days; (**B**) MTW starch after fermentation for 6, 8 and 10 days; (**C**) MTW starch after fermentation for 12, 14 and 16 days; (**D**) MTW starch after fermentation for 20, 24 and 28 days.(**S0**–**S11**) refer to MTW starch after fermentation for 0, 2, 4, 6, 8, 10, 12, 14, 16, 20, 24 and 28 days, respectively.

Fourier transform infrared spectroscopy (FTIR) is often used to characterize changes in starch functional groups and short-range ordered structures [44]. Figure 5 shows the Fourier infrared spectra of Millet starch in the 400~4000 cm^−1^ region after different fermentation times. The diffraction peaks of starch at 3600~3000 cm^−1^, 2950~2850 cm^−1^, 1656 cm^−1^ and 800~1300 cm^−1^ correspond to hydroxyl, H bond and C-H stretching, respectively. It can be seen in Figure 5 that millet starch and MTW starch at different fermentation stages have similar Fourier infrared spectra, indicating that fermentation did not produce new bonds or new functional groups. Generally, the short-range order degree (DO) and double-helix degree (DD) of starch were characterized by the absorbance ratio using Fourier infrared spectroscopy at 1047 and 1022 cm^−1^ and at 1022 and 995 cm^−1^ (DD) [45]. Table 2 shows that the DO of millet starch in different fermentation periods undergoes a large change, while the DD value shows a decreasing trend, indicating that the short-range ordered structure of fermented millet starch has undergone a complex change, which could be attributed to the change in the morphological structure, as observed with SEM and CLSM. The large starch granules appeared swollen, contained cavities, and had ruptured, disintegrated or even vanished during fermentation, while small starch granules only dispersed from the aggregates. These changes may lead to complex changes in the short-range ordered structure of MTW starch.

### 3.5. Pasting Properties

**Figure 6:**
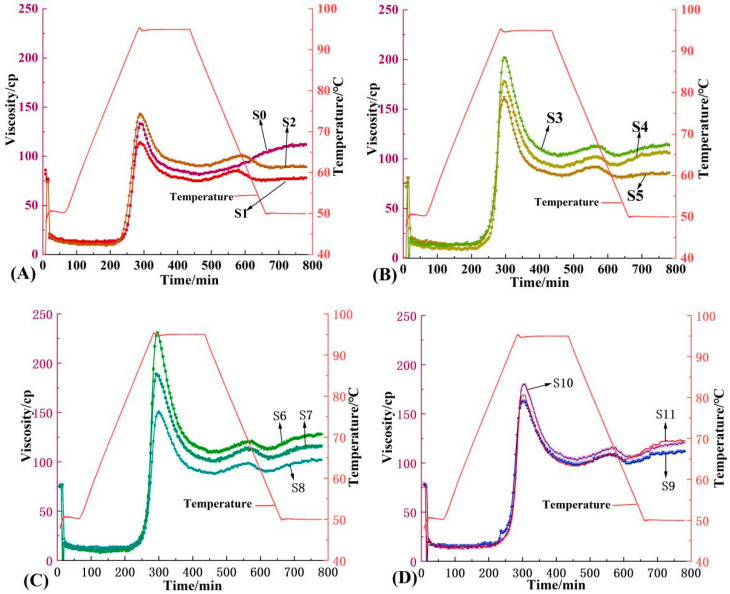
Viscosity curves of MTW starch on different fermentation days. Notes: (**A**) MTW starch after fermentation for 0, 2 and 4 days; (**B**) MTW starch after fermentation for 6, 8 and 10 days; (**C**) MTW starch after fermentation for 12, 14 and 16 days; (**D**) MTW starch after fermentation for 20, 24 and 28 days.(**S0**–**S11**) refer to MTW starch after fermentation for 0, 2, 4, 6, 8, 10, 12, 14, 16, 20, 24 and 28 days, respectively.

The gelatinization characteristic curves of millet and MTW starch at different fermentation stages are shown in Figure 6. Gelatinization parameters such as peak viscosity, trough viscosity and setback viscosity are shown in Table 3. It can be seen from Table 3 that the peak viscosity, trough viscosity, breakdown viscosity and final viscosity present change trends with a decrease, increase and then a decrease with the prolongation of the fermentation time. S6 had the highest peak viscosity, trough viscosity, breakdown viscosity and final viscosity. The decrease in peak viscosity may be caused by the acidic environment during fermentation [46]. It may also be due to the decrease in non-starch components such as proteins during fermentation, which causes starch granules to more easily absorb water and expand when gelatinizing. Lim et al. [47] showed that the decrease in protein content in rice flour could increase its peak viscosity.

The setback viscosity reflects the tendency of starch to recrystallize [48]. During fermentation, the setback viscosity first decreased and then increased, but it was lower than that of unfermented starch. Compared with natural starch, the fermentation process seems to delay the aging tendency of millet starch. With the prolongation of the fermentation time, the peak time and pasting temperature increased gradually, which was mainly due to the changes in the morphology and structure of different starch granules during fermentation. It may be that fermentation produces low-molecular-weight substances, resulting in the low swelling power of starch samples. As shown by the results of SEM and CLSM, with the prolongation of the fermentation time, the large starch granules, which are easy to gelatinize, gradually disintegrate, and the small starch granules, which are difficult to gelatinize, disperse from the aggregates and become the main part of the starch matrix, which leads to changes in the gelatinization characteristics.

## 4. Conclusions

In this study, changes in the millet starch structure and pasting properties during the preparation of MTW were investigated to understand the effects of fermentation on the quality of MTW. The study found that two sizes of starch granules existed in millet and MTW: large granules with diameters of 3 μm (A-granules) and small granules with diameters of about 1–2 μm wrapped in pomegranate-seed-like aggregates. During fermentation, large granules gradually lost the original morphological characteristics, whereas the size and shape of the small granules barely changed, only falling off the aggregates, indicating that the small granules cannot be fermented by microorganisms and endow MTW with good functional characteristics. With the prolongation of the fermentation time, the relative crystallinity and short-range ordered structures of starch in MTW undergo complex changes, which lead to an increase in the peak viscosity time and initial gelatinization temperature. The present results reveal that fermentation has a potentially beneficial effect on the quality of MTW.

## Figures and Tables

**Table 1:** Relative crystallinity (RC) of MTW starch in different fermentation periods.

Sample	S0	S1	S2	S3	S4	S5	S6	S7	S8	S9	S10	S11
RC/%	3.00	5.61	9.03	14.48	13.12	9.58	5.21	13.41	13.48	13.01	17.52	17.82

**Table 2:** Short-range order parameters of MTW starch on different fermentation days.

FTIR	S0	S1	S2	S3	S4	S5	S6	S7	S8	S9	S10	S11
R995/1022/DD	1.026	1.042	1.031	1.019	1.043	1.046	1.022	1.010	1.034	1.015	1.016	1.074
R1047/1022/ DO	1.030	1.026	1.025	1.018	1.028	1.041	1.008	1.011	1.025	1.008	1.010	1.048

**Table 3:** Pasting characteristic values of MTW starch on different fermentation days by RVA.

Sample	Peak Viscosity/cP	Trough Viscosity/cP	Breakdown Viscosity/cP	Final Viscosity/cP	Setback Viscosity/cP	Peak Time/s	Pasting Temperature/°C
S0	133	81	52	112	31	4.80	91.95
S1	114	75	39	78	3	4.80	91.98
S2	143	88	55	89	1	4.80	91.99
S3	162	81	81	86	5	4.93	92.00
S4	178	92	86	106	14	4.93	92.10
S5	202	103	99	114	11	4.93	92.10
S6	231	110	121	128	18	4.93	92.00
S7	189	101	88	116	15	4.87	92.00
S8	151	88	63	102	14	5.00	92.80
S9	163	99	64	112	13	5.00	92.85
S10	180	103	77	121	18	5.07	93.55
S11	169	97	72	123	26	5.00	93.60

## Data Availability

The data presented in this study are available on request from the corresponding author. The data are not publicly available, due to the request for funding scientific research projects.
